# Leber’s hereditary optic neuropathy with late disease onset: clinical and molecular characteristics of 20 patients

**DOI:** 10.1186/s13023-014-0158-9

**Published:** 2014-10-23

**Authors:** Konstantin Dimitriadis, Miriam Leonhardt, Patrick Yu-Wai-Man, Matthew Anthony Kirkman, Alex Korsten, Irenaeus F De Coo, Patrick Francis Chinnery, Thomas Klopstock

**Affiliations:** Department of Neurology, Friedrich-Baur-Institute, Ludwig-Maximilians-University, Munich, Germany; Welcome Trust Centre for Mitochondrial Research, Institute of Genetic Medicine, Newcastle University, Newcastle upon Tyne, UK; Departments of Neurology and Ophthalmology, Royal Victoria Infirmary, Newcastle upon Tyne, UK; Department of Neurology, Erasmus Medical Center, University Medical Center Rotterdam, Rotterdam, The Netherlands; DZNE-German Center for Neurodegenerative Diseases, Munich, Germany; Munich Cluster for Systems Neurology (SyNergy), 80336 Munich, Germany; German Network for Mitochondrial Disorders (mitoNET), Munich, Germany

**Keywords:** LHON, Late-onset LHON, Environmental factors

## Abstract

**Background:**

Leber’s hereditary optic neuropathy (LHON) is a mitochondrial disease that typically causes bilateral blindness in young men. Here we describe the clinical and molecular characteristics of 20 patients with disease onset after the age of 50 years (late onset-LHON).

**Methods:**

From a cohort of 251 affected and 277 unaffected LHON carriers, we identified 20 patients with onset of visual loss after the age of 50 years. Using structured questionnaires, data including basic demographic details, age of onset, progression of visual loss and severity as well as exposure to possible environmental triggers including alcohol, smoking and illicit drugs were retrospectively collected. Groups were compared using the Mann–Whitney-U-Test for two independent groups of sampled data.

**Results:**

The proportion of late onset-LHON in our cohort was 8% (20 patients, 15 males, 5 females). The mtDNA mutations m.11778G > A and m.3460G > A were found in 16 and 4 patients, respectively. Among 89 asymptomatic carriers above the age of 50 years (28 males, 61 females), the mtDNA mutations m.11778G > A, m.3460G > A and m.14484 T > C were found in 60, 12 and 17 carriers, respectively. Late onset-LHON patients had significantly higher mean cumulative tobacco and alcohol consumption compared with unaffected carriers. However, there was no significant difference between late onset- and typical LHON patients with regard to daily tobacco and weekly alcohol consumption before disease onset.

**Conclusion:**

As already shown for typical LHON, alcohol consumption and smoking are important trigger factors also for the late manifestation. LHON should be considered in the differential diagnosis of subacute blindness even in older patients.

## Background

Leber’s hereditary optic neuropathy (LHON) is a mitochondrial disease characterized by acute or subacute visual loss. The vast majority of patients (90%) carry one of three primary mitochondrial DNA (mtDNA) mutations: m.11778G > A, m.3460G > A or m.14484 T > C [1-3]. The prevalence of the disorder has been estimated at about 1 in 30,000 whereas the mutation carrier rate is estimated to be 1 in 350 [4-6].

LHON is generally perceived as a disease of young men, reflecting its male preponderance (80%) and its peak age of onset between the age of 15 and 30 years. However, cases with late onset of visual loss have been described [7-16].

Both the incomplete penetrance and the male predominance in LHON are not fully explained yet. It is considered as a multifactorial disease with complex interactions between genetic and environmental factors. In a previous study, we demonstrated that heavy smoking is a significant risk factor for disease manifestation [17].

Here we present the clinical and molecular characteristics of 20 patients with disease onset after the age of 50 years, and define this group as late onset-LHON.

## Subjects and methods

Our study cohort included 251 affected LHON patients and 277 asymptomatic mutation carriers harbouring one of the three primary mtDNA mutations (m.11778G > A, m.3460G > A or m.14484 T > C). Data were available on demographics, age of onset, progression of visual loss and severity, mutation type, and possible triggering factors for visual loss by using a standardized questionnaire). Data on visual function index (VF-14) scores for 402 subjects were also collected, as published previously [17,18]. From this cohort, we identified 20 patients with onset of LHON after the age of 50 years and 89 asymptomatic carriers aged >50 years. All subjects were of white Caucasian origin except one Asian individual. The respective Institutional Review Boards (Ludwig-Maximilians-University, Munich, Germany; Newcastle University, UK; and University Medical Center Rotterdam, The Netherlands) approved the study, and informed consent was obtained from each participant.

Data on possible environmental triggers like alcohol, smoking and illicit drugs were retrospectively collected. As described in our previous study [17], cumulative smoking was expressed in terms of ‘pack years’ (PY, number of cigarettes smoked per day by number of years smoking), whereas the maximum number of cigarettes consumed in a single day was defined as maximum smoking intensity. Similarly, alcohol consumption was expressed in ‘drink years’ (DY, units of alcohol per week multiplied by the number of years drinking). Maximum consumption of alcohol was defined as the highest number of units (U) of alcohol consumed in one week. In line with the previous study [17], only the consumption prior to the onset of vision loss was considered, both for smoking and alcohol consumption of affected individuals.

SPSSTM v.19 statistical software (Chicago, IL, USA) was used for statistical analysis. The rate of diseased carriers was calculated performing a lifetime distribution function F(t) = 1-S(t), were S is the survival function and t time, including censored cases. Groups (carriers with late onset-LHON vs. asymptomatic carriers >50 years old vs. LHON patients with onset of symptoms before age of 50) were compared using the Mann–Whitney-U-Test for two independent groups of sampled data.

## Results

20 out of 253 LHON patients were identified with a disease onset >50 years (8%). Similar to the complete study population, the male to female ratio among the affected 20 late onset patients was 3:1 (15 male and 5 female). The mtDNA mutations m.11778G > A and m.3460G > A were found in 16 (80%) and 4 (20%) patients, respectively. Among the 277 asymptomatic mutation carriers, 89 were above age 50 (32%), 28 being male and 61 female. The mtDNA mutations m.11778G > A, m.3460G > A and m.14484 T > C were found in 60 (67.4%), 12 (13.5%) and 17 (19.1%) of those asymptomatic carriers, respectively (Table 1). Except for one patient where information about alcohol and tobacco consumption was missing, all the other patients consumed alcohol, and 14 out of 19 patients smoked prior to becoming symptomatic. Four patients indicated to have had higher than average stress levels straight before symptoms started (Table 2).

**Table 1:** **Characteristics of study population**

	**Total population**		**Carriers <50 Years**		**Carriers ≥50 Years**	
	**Affected**	**Unaffected**	**Affected**	**Unaffected**	**Affected**	**Unaffected**
Mutation type						
m.11778G > A (%)	167 (66.0)	190 (68.6)	118 (66.3)	78 (66.7)	16 (80)	60 (67.4)
m.3460G > A (%)	53 (20.9)	49 (17.7)	31 (17.4)	24 (20.5)	4 (20)	12 (13.5)
m.14484 T > C (%)	33 (13.0)	38 (13.7)	29 (16.3)	15 (12.7)	0 (0)	17 (19.1)
Total	253	277	178	117	20	89
Sex N, (%)						
Male	194 (76.7)	92 (33.2)	134 (74.9)	32 (27.4)	15 (75.0)	28 (31.5)
Female	59 (23.3)	185 (66.8)	45 (25.1)	85 (72.6)	5 (25.0)	61 (68.5)
Male/Female ratio	3.3	0.5	3.0	0.37	3.0	0.46
Mean disease duration	15.9 (16.16)		10.71 (10.47)		10.5 (19.12)	
VF-14 score (SD)	25.1 (20.8)	97.3 (7.1)	25.14 (21.18)	98.01 (5.22)	18.06 (15.27)	96.45 (8.91)

**Table 2:** **Characteristics of late onset-LHON patients**

	**Age at onset**	**Sex**	**Mutation type**	Tobacco*	Alcohol*	**Other Triggers**
1	50	F	11778	+	+	-
2	50	F	11778	+	+	-
3	52	M	11778	+	+	-
4	53	M	11778	+	+	-
5	53	M	11778	+	+	+^1^
6	53	M	11778	+	+	-
7	53	M	11778	-	+	+^1^
8	53	M	11778	+	+	-
9	55	M	11778	+	+	-
10	56	M	11778	+	+	+^1^
11	56	F	11778	+	+	-
12	57	M	11778	+	+	-
13	58	F	3460	-	+	-
14	61	M	11778	-	+	-
15	62	M	3460	+	+	-
16	67	M	11778	-	+	-
17	74	F	3460	-	+	-
18	75	M	3460	+	+	-
19	76	M	11778	+	+	+^1^
20	75	M	11778	?	?	?

*Marked as positive only if consumption was prior to onset of disease.

^1^Patients indicated increased “stress” as a trigger.

As already shown in our previous study, the rate of diseased carriers per age was not influenced by the type of primary LHON mutation (lifetime distribution function, log rank p = 0.654). However, the rate of affected male individuals was significantly higher (lifetime distribution function, log rank p < 0.000).

There was no significant difference in the mean duration of disease (time from onset) in patients with late onset-LHON versus patients with onset <50 years (8.9+/−9.02 years versus 6.79+/−11.32, p = 0.123). As expected and in line with our previous study, the mean VF-14 score for affected patients with late onset-LHON was significantly lower than that of unaffected carriers over 50 years old (18.06+/−15.27 versus 96.45+/−8.91, p < 0.000). Comparing the VF-14 scores of late onset-LHON patients with symptomatic carriers with onset <50 years showed a trend to lower scores for the older patients (18.06+/−15.27 versus 25.14+/−21.18, p = 0.144). Within the late onset-LHON cohort, patients carrying the m.3460G > A mutation had lower scores than patients carrying the m.11778 G > A mutation, but the result was not significant (13.89+/−15.43 versus 19.26+/−15.59, p = 0.490).

Late onset-LHON patients had significantly higher mean cumulative tobacco consumption compared with unaffected carriers above age 50 years (23.4+/−27.5 pack years (PY), versus 9.3+/−13.9 PY, p = 0.018) and there was a trend to higher mean maximum intensity of smoking (19.33+/−17.25 cigarettes per day versus 11.3+/−14.56 cigarettes per day, p = 0.059). Concerning alcohol, symptomatic carriers had both a significant higher cumulative consumption and mean maximum intensity of drinking (137.2+/−108.2 UY versus 62.2+/−87.0 UY, p = 0.012 and 99.86+/−255.71 U/week versus 14.61+/−22.06 U/week, p < 0.000) (Table 3A).

**Table 3:** **Tobacco and alcohol consumption among late onset-LHON patients, unaffected carriers and “typical” LHON patients**

**A) Levels of tobacco and alcohol consumption in late onset-LHON patients vs. unaffected carriers over 50 years old**			
	**Affected N =20**	**Unaffected N =89**	**p value**
	Mean (SD)	Mean (SD)	
Cumulative smoking [PY]	23.36 (27.49)	9.25 (13.92)	0.018
Maximum smoking consumption [CPD]	19.33 (17.25)	11.3 (14.56)	0.059
Cumulative alcohol [UY]	137.18 (108.17)	62.23 (87.04)	0.012
Maximum alcohol consumption [UPW]	99.86 (255.71)	14.61 (22.06)	0.000
**B) Tobacco and alcohol consumption in late onset- vs. “typical” LHON patients**			
	**Affected N =20 > 50 Years**	**Affected N =178 < 50 Years**	
Daily cigarette consumption [CPD]	13.63 (14.48)	10.03 (10.43)	0.619
Weekly alcohol consumption [UPW]	27.78 (29.14)	18.09 (19.47)	0.284

PY = pack years, CPD = cigarettes per day, UY = unit years, UPW = units per week.

3 A) Cumulative and maximum smoking and alcohol consumption in late onset-LHON patients as compared to unaffected carriers over 50 years B) daily cigarette and alcohol consumption before disease onset in late onset- as compared to ‘typical’ LHON patients.

Expectedly, due to longer exposure, life-time cumulative smoking and alcohol consumption were significantly higher in late-onset as compared to typical LHON patients (23.4+/−27.5 pack years (PY), versus 4.9+/−8.01 PY, p < 0.000; 137.2+/−108.2 UY versus 33.3+/−56.0 UY, p = 0.001). However, there was no significant difference with regard to daily tobacco and weekly alcohol consumption before disease onset (13.6+/−14.5 cigarettes/day versus 10.3+/−10.4 cigarettes/day, p = 0.619 and 27.8+/−29.1 U/week versus 18.1+/− 19.5 U/week, p = 0.284) (Table 3B). This was also true when the analysis was restricted to the 14 late-onset LHON and 89 typical LHON cases that had exposure to both alcohol and tobacco (data not shown).

## Discussion

Although most affected individuals with LHON lose vision in the second or third decade of life, the disease is not restricted to young adults. Here we describe a cohort of patients with onset after the age of 50 years (late onset-LHON). Late onset occurred in 20 of 251 LHON patients in our cohort, corresponding to 8% of patients. While all previously reported cases of late onset in LHON were males, the sex ratio in our study is similar to typical disease in young adults with a male to female ratio of about 3:1. Likewise, we found no difference between typical and late-onset LHON in the distribution of the causative mtDNA mutations and in the severity of the disease as measured using the VF-14 index.

The triggering factors for visual loss in patients harboring one of the typical mutations for LHON are not fully known. Apart from alcohol and smoking, for which a correlation was recently demonstrated [17], many other triggers have been described in single case reports, such as lack of oestrogens, trauma, antiretroviral therapy, HIV, anaemia, cyanide and carbon monoxide intoxication [9,19-23].

Recent studies provided some molecular evidence that estrogens activate the antioxidant enzyme superoxide dismutase 2 and also the mitochondrial biogenesis and thus play a role in maintaining vision in female LHON carriers [24]. In one female with a 11778G > A mutation the natural absence of estrogens at a young age (because of a Perrault syndrome) is discussed as a possible reason for the severe optic atrophy with early onset [25]. While our data do not allow for conclusions on protective effects of estrogens, the similar male/female ratios both in late onset vs. typical LHON patients and in unaffected mutation carriers above vs. below age 50 years argue clearly against post-menopause as a manifestation factor of the disease.

As already shown for typical LHON, alcohol consumption and smoking are important risk factors for disease conversion among patients with late onset-LHON. In the literature, there was one 67-year-old patient who lost his vision after blood loss due to hemorrhage from multiple small bowel arteriovenous malformations and a second one with a long history of HIV and zidovudine treatment prior to onset of disease [9,19]. Recently a case series of three late onset-LHON patients with raised intraocular pressure as a potential risk factor triggering visual loss were described [26]. Besides alcohol consumption and smoking, no other secondary factors influencing disease expression were elucidated in late-onset LHON patients reported in this study or in the literature. The finding that life-time cumulative smoking and alcohol consumption are significantly higher in late-onset as compared to younger LHON patients is rather self-evident and does not provide any explanation for the later disease manifestation. More interestingly, the daily tobacco and weekly alcohol consumption before disease onset were not significantly different between late-onset and typical LHON patients. The fact that these pre-manifestation exposure rates to alcohol and tobacco as well as mutation profile and gender distribution are very similar in late onset-LHON and in the younger cases indicates that environmental factors are only partly explaining the variable penetrance of the disease.

## Conclusion

In conclusion, alcohol consumption and smoking are important trigger factors not only for typical LHON but also for late-onset LHON. Moreover, this study shows that LHON should be considered in the differential diagnosis of subacute blindness even in older patients.
